# Characterisation of community-acquired *Staphylococcus aureus* causing skin and soft tissue infections in a children's hospital in Shanghai, China

**DOI:** 10.1017/S0950268819002127

**Published:** 2019-12-13

**Authors:** Xing Wang, Yanyun Shen, Weichun Huang, Yun Zhou

**Affiliations:** 1 Department of Laboratory Medicine, Shanghai Children's Medical Centre, Shanghai Jiaotong University School of Medicine, Shanghai, P. R. China; 2 Department of Dermatology, Huashan Hospital, Fudan University, Shanghai, P. R. China; 3 Department of Critical Care Medicine, Huashan Hospital, Fudan University, Shanghai, P. R. China

**Keywords:** Molecular epidemiology, paediatrics, skin and soft tissue infection, *Staphylococcus aureus*

## Abstract

Community-acquired *Staphylococcus aureus* is a major pathogen responsible for skin and soft tissue infections (SSTIs). This study aimed to investigate the prevalence and molecular characteristics of community-acquired *S. aureus* isolates recovered from paediatric patients with SSTIs in Shanghai, China. Between January 2015 and January 2018, 91 community-acquired *S. aureus* isolates were characterised by antibiotic susceptibility, multilocus sequence typing (ST), staphylococcal protein A gene (*spa*) type and virulence genes. Methicillin-resistant *S. aureus* (MRSA) strains were also characterised by staphylococcal cassette chromosome *mec* (SCC*mec*) type. Forty-one (45.1%) *S. aureus* isolates were MRSA. ST59 (33.0%, 30/91) was the most common sequence type, and t437 (18.7%, 17/91) was the most common *spa* type. SCC*mec* IV and V accounted for 61.0% and 34.1% of all MRSA isolates, respectively. Each isolate carried at least six virulence genes. The positive rates of Panton-Valentine leukocidin genes among all *S. aureus*, MRSA and methicillin-susceptible *S. aureus* isolates were 30.8% (28/91), 39.0% (16/41) and 24% (12/50), respectively. The prevalence of community-associated MRSA was surprisingly high among children with community-acquired SSTIs in Shanghai. ST59-t437 was the most prevalent community-acquired *S. aureus* clone causing SSTIs.

## Introduction

*Staphylococcus aureus* is a major cause of skin and soft tissue infections (SSTIs) worldwide [1, 2]. Community-associated methicillin-resistant *S. aureus* (CA-MRSA) has been a global concern since it emerged in the 1990s. Although CA-MRSA strains can cause nosocomial infections, and healthcare-associated MRSA (HA-MRSA) strains do circulate in the community, CA-MRSA is epidemiologically, clinically and genetically different from HA-MRSA [3]. CA-MRSA infections tend to occur in otherwise healthy younger patients with SSTIs; such strains may be susceptible to a wider range of antibiotics, and usually harbour the staphylococcal cassette chromosome *mec* (SCC*mec*) types IV or V. The most prevalent clones of CA-MRSA have relatively unique geographic distributions, for instance, sequence type 8 (ST8, USA300) clones have mainly been reported in the USA, ST80 in Europe, ST30 in western Pacific area and ST59 in the Asia-Pacific region [4]. The global epidemic of CA-MRSA continues to evolve, and the potential shift of epidemic clones at the local, national and international levels is of great interest.

Clinicians and researchers are primarily concerned with CA-MRSA, but community-associated methicillin-susceptible *S. aureus* (CA-MSSA) should not be overlooked as they are the most common causative agent of SSTIs in North America, Latin America and Europe [5], as well as in Japan [6]. It is hypothesised that MRSA strains probably originate from MSSA strains which acquire the SCC*mec* determinants. Therefore, epidemiological surveillance of CA-MSSA is as important as for CA-MRSA in order for effective control of infection and transmission in the affected communities.

Currently, limited molecular characterisation data of *S. aureus* strains isolated from community-acquired SSTIs are available in mainland China, and particularly regarding paediatric patients in Shanghai. The aim of the present study was to determine the prevalence and molecular characteristics of *S*. *aureus* isolates recovered from paediatric patients with community-acquired SSTIs in Shanghai, the most developed city in eastern China.

## Methods

### Ethics

The Ethics Committee of Shanghai Children's Medical Centre exempted this study from review as it focused on bacterial isolates alone. The review board waived the need for written consent, and guardians of patients provided oral informed consent.

### Bacterial isolates

This retrospective study included *S*. *aureus* isolates recovered from all paediatric patients with community-acquired SSTIs between January 2015 and January 2018 in a university teaching hospital in Shanghai (Shanghai Children's Medical Centre, one of the largest and best paediatric hospitals in China with 604 beds and approximately 22000 hospital discharges per year). SSTIs were identified by the presence of redness, swelling and localised pain. In order to eliminate possible culture contamination or bacterial colonisation, only those isolates from febrile patients (axillary temperature ≥37.5 °C or rectal temperature ≥38 °C) with increased white blood cell count were included. Community-acquired *S. aureus* was defined as an isolate from either an outpatient, or an inpatient within 48 h of hospitalisation, who had no medical history of *S. aureus* infection/colonisation, hospitalisation, surgery, dialysis or indwelling catheter in the past year. The first *S*. *aureus* isolate obtained from each patient was selected for analysis.

### Antimicrobial susceptibility testing

All isolates were subjected to antimicrobial susceptibility testing using the bioMérieux Vitek 2 system according to the manufacturer's instructions. Results were interpreted following the recommendations and definitions of the Clinical and Laboratory Standards Institute (CLSI, 2017). The following 17 drugs were tested: penicillin, oxacillin, cefoxitin, erythromycin, clindamycin, gentamicin, moxifloxacin, levofloxacin, ciprofloxacin, tetracycline, rifampicin, trimethoprim-sulfamethoxazole, quinupristin/dalfopristin, nitrofurantoin, vancomycin, linezolid and tigecycline. *S. aureus* ATCC 29213 was used as the quality control strain. Criteria for defining multidrug-resistant *S. aureus* are: (i) an MRSA and (ii) an MSSA not susceptible to at least one agent in three or more antimicrobial categories [7].

### Multilocus sequence typing (MLST), staphylococcal protein A gene (spa) and SCC*mec* typing

All isolates were screened according to protocols described on the *S. aureus* MLST website (https://pubmlst.org/saureus/) and the *S. aureus spa* type database (https://www.spaserver.ridom.de/). Clonal complexes (CCs) were analysed using eBURST on the basis of related STs. All MRSA strains were subjected to SCC*mec* typing as reported previously [8].

### Detection of virulence genes

The presence or absence of the following 33 virulence genes was tested in all isolates as previously described [9–11]: staphylococcal enterotoxin genes (*sea*, *seb*, *sec, sed, see, seg, seh, sei*, *sej*, *sek*, *sel*, *sem*, *sen*, *seo*, *sep* and *seq*), toxic shock syndrome toxin gene (*tsst-1*), arginine catabolic mobile gene (*arcA*), exfoliative toxin genes (*eta* and *etb*), genes encoding Panton-Valentine leukocidin (PVL) and other leukocidins (*lukF/S-PV*, *lukE* and *lukM*), hemolysin genes (*hla*, *hlb*, *hld*, *hlg* and *hlg2*) and adhesin genes (*clfA*, *icaA*, *sdrC*, *sdrD* and *sdrE*).

### Statistical analysis

Statistical analyses were performed by Stata software (version 10.1/SE, Stata Corp, College Station, TX, USA) via the *χ*^2^ test and Fisher's exact test. Statistical significance was set at *P* value ⩽ 0.05.

## Results

### Clinical features

A total of 91 children aged 7 days to 17 years with community-acquired *S. aureus* SSTIs were identified. Of these, 47.3% (43/91) were male, 62.3% (57/91) were inpatients, 13.2% (12/91) were neonates, 41.8% (38/91) were under 1-year old, 20.9% (19/91) were 1 to 3 years old, 31.9% (29/91) were 4 to 10 years old and 5.5% (5/91) were teenagers.

Abscess (68.1%, 62/91) was the most common type of infection, followed by vulvitis (11.0%, 10/91), otitis externa (7.7%, 7/91), infection of eyelid and lacrimal apparatus (4.4%, 4/91) and other presentations (cellulitis, neonatal omphalitis, paronychia and wound infection). Among the 57 inpatients, 54 (94.7%) underwent surgical intervention (drainage/debridement); only 2 of 34 (5.9%) outpatients required incision and drainage.

Fifty (54.9%) children were infected with MSSA, the remainder with MRSA. Among the MSSA patients 52% (26/50) were admitted to hospital, compared with 75.6% (31/41) MRSA patients who required hospitalisation.

### Molecular typing

**Fig. 1.**
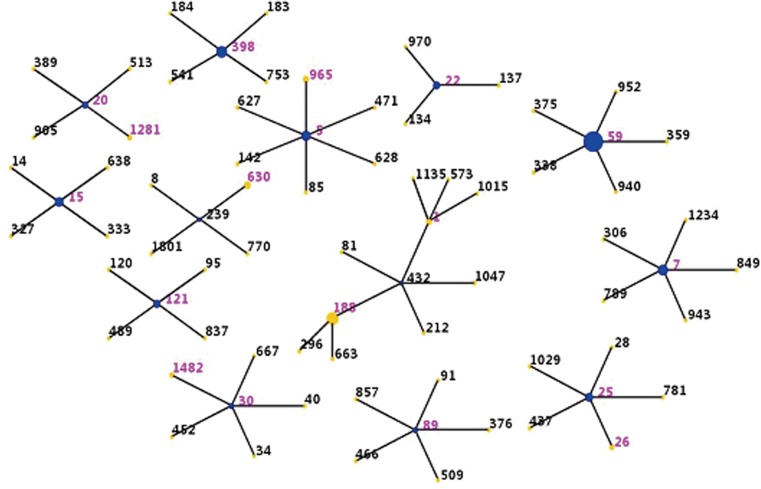
Distribution of sequence types in the CCs. The purple numbers represent 18 sequence types found in 91 community-associated *S. aureus* strains isolated from SSTI samples. Sequence types belonging to the same cluster are connected by a line. Each circle size is proportional to the number of isolates within the sequence type.

Eighteen distinct STs were identified by MLST (Table 1); ST59 was the most common (27 MRSA and 3 MSSA isolates). All strains were clustered by eBURST into 13 CCs (CC59, CC188, CC398, CC7, CC5, CC15, CC25, CC22, CC121, CC8, CC20, CC1 and CC30) and 1 singleton (ST89) (Fig. 1).

**Table 1.** Molecular typing of 91 community-acquired *S. aureus* isolates from paediatric patients with SSTIs

Clonal complex (*n*, %)	Sequence type (*n*, %)	*spa* type (*n*, %)	SCC*mec* type (*n*)
CC59 (30, 33.0)	ST59 (30, 33.0)	t437 (17, 18.7)	IV (8), V (8)
		t172 (5, 5.5)	IV (3)
		t441 (3, 3.3)	IV (3)
		t163 (1, 1.1)	IV (1)
		t1151 (1, 1.1)	IV (1)
		t3517 (1, 1.1)	IV (1)
		t3736 (1, 1.1)	IV (1)
		NT (1, 1.1)	IV (1)
CC188 (11, 12.1)	ST188 (11, 12.1)	t189 (11, 12.1)	/
CC398 (8, 8.8)	ST398 (8, 8.8)	t034 (7, 7.7)	V (4)
		t571 (1, 1.1)	/
CC5 (8, 8.8)	ST5 (6, 6.6)	t002 (2, 2.2)	IV (1)
		t688 (1, 1.1)	IV (1)
		t045 (1, 1.1)	/
		t548 (1, 1.1)	/
		t4336 (1, 1.1)	/
	ST965 (2, 2.2)	t062 (2, 2.2)	IV (1)
CC7 (7, 7.7)	ST7 (7, 7.7)	t091 (4, 4.4)	V (1)
		t803 (1, 1.1)	/
		t1685 (1, 1.1)	/
		t1943 (1, 1.1)	/
CC15 (5, 5.5)	ST15 (5, 5.5)	t084 (2, 2.2)	V (1)
		t346 (2, 2.2)	/
		t1509 (1, 1.1)	/
CC25 (5, 5.5)	ST25 (4, 4.4)	t087 (2, 2.2)	/
		t078 (1, 1.1)	III (1)
		t280 (1, 1.1)	/
	ST26 (1, 1.1)	t258 (1, 1.1)	/
CC22 (3, 3.3)	ST22 (3, 3.3)	t309 (2, 2.2)	/
		t5763 (1, 1.1)	/
CC121 (3, 3.3)	ST121 (3, 3.3)	t159 (1, 1.1)	/
		t2019 (1, 1.1)	/
		NT (1, 1.1)	/
CC8 (3, 3.3)	ST630 (3, 3.3)	t377 (1, 1.1)	/
		t4549 (1, 1.1)	II (1)
		t4047 (1, 1.1)	/
CC20 (3, 3.3)	ST20 (2, 2.2)	t164 (2, 2.2)	/
	ST1281 (1, 1.1)	t3076 (1, 1.1)	/
CC1 (2, 2.2)	ST1 (2, 2.2)	t127 (1, 1.1)	IV (1)
		t12303 (1, 1.1)	IV (1)
CC30 (2, 2.2)	ST30 (1, 1.1)	t019 (1, 1.1)	IV (1)
	ST1482 (1, 1.1)	t6119 (1, 1.1)	/
	ST89 (1, 1.1)	t375 (1, 1.1)	/

The genetic diversity of all isolates was confirmed by the identification of 41 *spa* types (Table 1); t437 (18.7%, 17/91: 16 MRSA, 1 MSSA) was the predominant *spa* type, followed by t189 (12.1%, 11/91), t034 (7.7%, 7/91), t172 (5.5%, 5/91), t091 (4.4%, 4/91) and t441 (3.3%, 3/91). Other *spa* types were each represented by fewer than three isolates. All t437 strains belonged to ST59, and the majority (56.7%, 17/30) of strains in this ST were *spa* type t437. All t189 strains fell in ST188, and vice versa.

SCC*mec* typing revealed 4 types (types II, III, IV and V) among the MRSA isolates (Table 1) with type IV being the most common accounting for 25 of 41 isolates (61.0%) followed by type V (34.1%, 14/41). Types II and III were each represented by single isolates.

### Antimicrobial susceptibility profiles

The antimicrobial resistance profiles of isolates classified according to MLST are listed in Table 2. All were susceptible to quinupristin/dalfopristin, nitrofurantoin, vancomycin, linezolid and tigecycline. The vast majority of isolates (94.5%) were resistant to penicillin, 61.5% to erythromycin, 58.2% to clindamycin and 45.1% to oxacillin and cefoxitin. Among the MSSA isolates, 38% (19/50) were multidrug-resistant, and 24.4% (10/41) of the MRSA were resistant to ≤1 antimicrobial besides penicillin, oxacillin and cefoxitin.

**Table 2.** Antimicrobial susceptibility profiles of 91 community-acquired *S. aureus* isolates from paediatric patients with SSTIs

Sequence type (*n*)	Antimicrobial resistance (%)											
	P	OXA	FOX	E	DA^a^	GM	MXF	LEV	CIP	TET	RIF	SXT
Total (91)	94.5	45.1	45.1	61.5	58.2	4.4	1.1	1.1	3.3	22.0	2.2	11.0
ST59 (30)	100	90	90	76.7	76.7	0	0	0	0	40	0	0
Non-ST59 (61)	91.8	23.0	23.0	70.5	49.2	6.6	1.6	1.6	4.9	13.1	3.3	16.4
ST188 (11)	63.6	0	0	18.2	9.1	0	0	0	0	0	9.1	0
ST398 (8)	87.5	50	50	62.5	62.5	25	0	0	0	12.5	0	25
ST7 (7)	100	14.3	14.3	57.1	42.9	0	0	0	14.3	42.9	0	14.3
ST5 (6)	100	33.3	33.3	66.7	83.3	16.7	0	0	16.7	33.3	0	50
ST15 (5)	100	20	20	60	40	0	0	0	0	20	0	0
ST25 (4)	100	25	25	100	100	0	0	0	0	0	0	25
ST22 (3)	100	0	0	100	66.7	0	0	0	0	0	0	33.3
ST121 (3)	100	0	0	66.7	66.7	0	0	0	0	0	0	33.3
ST630 (3)	100	33.3	33.3	0	0	0	0	0	0	33.3	0	0
Other STs (11)^b^	100	36.4	36.4	54.5	54.5	9.1	9.1	9.1	9.1	0	9.1	9.1
*P* value^c^	0.11	<0.001	<0.001	<0.001	0.012	0.15	0.48	0.48	0.22	0.004	0.32	0.019

P, penicillin; OXA, oxacillin; FOX, cefoxitin; E, erythromycin; DA, clindamycin; GM, gentamicin; MXF, moxifloxacin; LEV, levofloxacin; CIP, ciprofloxacin; TET, tetracycline; RIF, rifampicin; SXT, trimethoprim-sulfamethoxazole.

a Inducible clindamycin resistance was performed with the bioMérieux Vitek 2 system.

b Other STs included ST965, ST26, ST20, ST1281, ST1, ST30, ST1482 and ST89.

c The resistance rates of antimicrobials were compared between ST59 and non-ST59 isolates.

### Virulence gene profiles

Table 3 shows that each isolate carried at least six virulence genes, and 72 (79.1%) isolates were positive for ≥10 genes. All isolates carried *hla*, *hld*, *icaA* and *clfA* but none was positive for *lukM* and *arcA* genes. PVL genes were identified in 30.8% (28/91) of all isolates, with ST59 (50.0%, 14/28) being predominant. Twelve (24%, 12/50) MSSA and 16 (39.0%, 16/41) MRSA were PVL-positive (24% *vs.* 39.0%, *P* = 0.122). Twenty-two (78.6%, 22/28) patients who were infected with PVL-positive strains and 34 (54.0%, 34/63) patients who were infected with PVL-negative strains underwent surgical intervention (78.6% *vs.* 54.0%, *P* = 0.026).

**Table 3.** Virulence gene profiles of 91 community-acquired *S. aureus* isolates from paediatric patients with SSTIs

Virulence gene	Gene carrying rate (%)			*P* value^a^
	*S. aureus* (*n* = 91)	ST59 (*n* = 30)	Non-ST59 (*n* = 61)	
*hlb*	44.0	93.3	19.7	<0.001
*hlg*	22.0	3.3	31.1	0.0026
*hlg2*	81.3	100	72.1	0.0013
*sdrC*	93.4	100	90.2	0.08
*sdrD*	45.2	13.3	60.7	<0.001
*sdrE*	81.3	100	72.1	0.0013
*lukF/S-PV*	30.8	46.7	23.0	0.02
*lukE*	69.2	36.7	85.2	<0.001
*tsst-1*	11.0	0	16.4	0.019
*eta*	3.3	3.3	3.3	0.99
*etb*	1.1	0	1.1	0.48
*sea*	17.6	26.7	13.1	0.11
*seb*	46.2	90.0	23.0	<0.001
*sec*	13.2	0	19.7	0.009
*sed*	11.0	3.3	14.8	0.1
*see*	5.5	0	8.2	0.11
*seg*	38.5	13.3	50.8	<0.001
*seh*	3.3	0	4.9	0.21
*sei*	37.4	13.3	49.2	<0.001
*sej*	13.2	3.3	18.0	0.051
*sek*	35.2	90	6.6	<0.001
*sel*	26.4	13.3	32.8	0.048
*sem*	38.5	16.7	49.2	0.003
*sen*	37.4	13.3	49.2	<0.001
*seo*	15.4	3.3	21.3	0.025
*sep*	15.4	6.7	19.7	0.106
*seq*	36.3	90	8.2	<0.001

a The positive rates of virulence genes were compared between ST59 and non-ST59 isolates.

### Molecular characteristics of the prevalent clone ST59

Compared with strains of other sequence types, ST59 were associated with higher resistance rates to oxacillin, cefoxitin, erythromycin, clindamycin and tetracycline, but with lower resistance to trimethoprim-sulfamethoxazole (Table 2). Likewise the presence of PVL genes among ST59 isolates was higher than for non-ST59 clones. Besides *pvl*, only six virulence genes: 2 encoding for haemolysin (*hlb* and *hlg2*), one adhesin (*sdrE*) and three enterotoxin genes (*seb*, *sek* and *seq*), were more common among ST59 isolates, while *hlg*, *sdrD*, *lukE*, *tsst*-*1* and the enterotoxin genes (*sec*, *seg*, *sei*, *sel*, *sem*, *sen* and *seo*), were more common among non-ST59 isolates (Table 3).

## Discussion

The proportion of CA-MRSA infections globally has increased significantly during the past 30 years. The prevalence of CA-MRSA SSTIs was reported to be 57% in the US in 2004 [12], but there was no report of such infections in mainland China before 2009. A few studies reported that CA-MRSA accounted for 4.0% of community-acquired *S*. *aureus* SSTIs in Beijing (northern China, isolates collected from children between 2008 and 2009), 3.4% in Guangzhou (southern China, 2006–2011, adults and children) and 10.3% in Wuhan (central China, 2011–2013, adults and children) [13–15]. Likewise a multicentre study (2009–2011, adults and children) found a low prevalence of CA-MRSA SSTIs ranging from 1.3% in northern China to 6.1% in southwestern China, and 3.8% in a children's hospital in Shanghai [16]. In contrast, we found the prevalence of CA-MRSA SSTIs in the present study to be surprisingly high (45.1%, 41/91). Between July 2012 and December 2013 in Shanghai Children's Medical Centre, only 3 of 13 patients (23.1%) were diagnosed as CA-MRSA SSTIs [17]; this rate rose to 42.1% (8/19) in 2014 (unpublished data), reaching 45.1% in the series of patients reported here. No statistically significant difference between the proportions of the initial and current was evident. Nevertheless, the data do indicate a marked increase in the total number of these infections since 2014. Although there may be regional variations, differences between adults and children and a data bias due to of small sample size, our findings indicate that the proportion of CA-MRSA SSTIs has increased rapidly in China.

A single-centre study of children with SSTIs in Beijing found ST59-SCC*mec* IV-*spa* t437 to be the major clone among CA-MRSA isolates, but no predominant *spa* type was evident for CA-MSSA isolates [13]. Likewise, a prospective surveillance study of community-acquired SSTIs conducted in 23 hospitals over a 24-month period in China, revealed that ST121 was the predominant CA-MRSA clone followed by ST59 [16]. Moreover, two other studies from China reported that the livestock-associated ST398 was the most prevalent PVL-positive clone of MSSA causing SSTIs [18, 19]. In our study, ST59-t437 was the major CA-MRSA clone, and ST188-t189 the most prevalent among CA-MSSA. Overall, ST59-t437 was the most common clone among community-acquired *S*. *aureus* strains isolated from paediatric SSTI patients in Shanghai. Antimicrobial resistance rather than virulence-associated genes may contribute to the spread of ST59 clone among children. As Shanghai has a highly mobile population, further studies monitoring the genotype distribution and dynamics of community-acquired *S*. *aureus* strains would be of value.

Testing for inducible clindamycin resistance was performed with the Vitek 2 system in this study. As this system has been associated with false susceptible results with for this agent [20], the actual rate of clindamycin resistance may be higher than the 58.2% observed. However, we did not focus on clindamycin resistance as neither lincosamides nor macrolides are used for empiric or first-line treatment for SSTIs in Shanghai. The high prevalence of resistance to both erythromycin and clindamycin in our series of isolates may be associated with overuse of azithromycin in paediatric patients with acute upper respiratory infection in China, but formal evidence for this is lacking.

PVL, a bicomponent pore-forming leukotoxin inducing leukocyte destruction and tissue necrosis, is more frequently detected in *S. aureus* isolates associated with SSTIs and in community-acquired strains [21, 22], and the determinant genes have been reported to be more common in MRSA than in MSSA [23, 24]. The prevalence of PVL-positive strains causing SSTIs in China was reported to range from 9.8% (5/51) to 71.4% (10/14) among CA-MRSA isolates, and from 4.2% (14/337) to 41.5% (66/159) among CA-MSSA isolates [13, 16, 18]. Our findings for rates of PVL genes among both CA-MRSA and CA-MSSA are consistent with previous studies with a similar prevalence of PVL genes in both groups. The association of PVL with an enhanced inflammatory response and pus-forming lesions that often require surgical intervention is well documented [18, 25, 26]. Our patients with PVL-positive strains also had a higher surgical intervention rate; however, among these 56 patients the majority (60.7%, 34/56) were infected with PVL-negative trains. The actual correlations between PVL and the pathogenesis of different SSTI clinical presentations warrant further study.

This study has some limitations. Although patients came from all over China, this is a single-centre study with a relatively small sample size due to the lack of wider microbiological screening. Nevertheless, as our medical centre is ranked within the top three best and top five best paediatric hospitals in the region, and China as a whole, we consider that our findings are likely to be representative of children both regionally and nationally. In addition, as only isolates obtained from SSTI patients with fever were included, and hence classified as complicated infection [2], our results may not necessarily apply to uncomplicated SSTIs, which are generally treated in community or district hospitals.

In conclusion, this study revealed that the prevalence of CA-MRSA was surprisingly high among children with community-acquired SSTIs in Shanghai, China. Clones ST59-t437 and ST188-t189 were most representative of CA-MRSA and CA-MSSA respectively; overall, ST59-t437 was the most prevalent among community-acquired SSTIs.
